# Late Onset Cobalamin Disorder and Hemolytic Uremic Syndrome: A Rare Cause of Nephrotic Syndrome

**DOI:** 10.1155/2017/2794060

**Published:** 2017-08-01

**Authors:** Gianluigi Ardissino, Michela Perrone, Francesca Tel, Sara Testa, Amelia Morrone, Ilaria Possenti, Francesco Tagliaferri, Robertino Dilena, Francesca Menni

**Affiliations:** ^1^Center for HUS Prevention Control and Management, Fondazione IRCCS Ca' Granda Osp. Maggiore Policlinico, Milan, Italy; ^2^Paediatric Neurology Unit and Laboratories, Neuroscience Department, Meyer Children's Hospital, Florence, Italy; ^3^Neuroscience, Psychology, Pharmacology and Child Health Department, University of Florence, Florence, Italy; ^4^Pediatric Unit, Pediatric Hospital C. Arrigo, Alessandria, Italy; ^5^Pediatric Unit, Fondazione IRCCS Ca' Granda Osp. Maggiore Policlinico, Milan, Italy; ^6^UOC Neurophysiology, Fondazione IRCCS Ca' Granda Osp. Maggiore Policlinico, Milan, Italy

## Abstract

Hemolytic uremic syndrome (HUS) is an unrare and severe thrombotic microangiopathy (TMA) caused by several pathogenetic mechanisms among which Shiga toxin-producing* Escherichia coli* infections and complement dysregulation are the most common. However, very rarely and particularly in neonates and infants, disorders of cobalamin metabolism (CblC) can present with or be complicated by TMA. Herein we describe a case of atypical HUS (aHUS) related to CblC disease which first presented in a previously healthy boy at age of 13.6 years. The clinical picture was initially dominated by nephrotic range proteinuria and severe hypertension followed by renal failure. The specific treatment with high dose of hydroxycobalamin rapidly obtained the remission of TMA and the complete recovery of renal function. We conclude that plasma homocysteine and methionine determinations together with urine organic acid analysis should be included in the diagnostic work-up of any patient with TMA and/or nephrotic syndrome regardless of age.

## 1. Introduction

Atypical hemolytic uremic syndrome (aHUS) is a life-threatening disease characterised by thrombotic microangiopathy (TMA) that is mainly due to Shiga toxin-producing* Escherichia coli* infections and uncontrolled complement activation, although it may rarely be due to other causes, such as disorders of intracellular cobalamin (cbl) metabolism [1]. Methylmalonic aciduria and homocystinuria, cobalamin C type (cblC) (OMIM 277400) is likely the most common of such disorders and is caused by biallelic mutations in the* MMACHC* gene (Lerner-Ellis et al. 2006). cblC-related TMA has been almost mainly reported in neonates and infants [2–8], but we have recently encountered a case that developed it at the very unusual age of 13.6 years. Its clinical presentation was dominated by symptoms of nephrotic syndrome and hypertension that initially responded to eculizumab treatment.

## 2. Case Presentation

A Caucasian son of unrelated parents was referred to our centre because of severe hypertension, nephrotic range proteinuria, microhematuria, acute kidney injury, and signs of TMA. His family history was unremarkable for kidney diseases and his own previous medical history was uneventful until the age of 8 years, when he experienced an episode of generalised seizure in apyrexia that was not followed by any relevant event and did not require any specific treatment.

One month before the acute episode, the boy suffered from severe headache and recurrent emesis associated with a weight loss (from 47 to 43 kg) for which he was admitted to another hospital. Severe arterial hypertension was detected (150/110 mmHg) and laboratory tests revealed nephrotic range proteinuria (5 gr/day), associated with signs of kidney injury: serum creatinine 1.3 mg/dL, microscopic hematuria together with mild thrombocytopenia (121,000/mm^3^) and hemolytic anemia (hemoglobin 10.2 g/dL, lactic dehydrogenase (LDH) 894 U/L, and undetectable haptoglobin). Renal ultrasonography revealed enlarged, hyperechogenic kidneys with reduced corticomedullary differentiation, and echocardiography showed left ventricular hypertrophy and a slightly enlarged aortic bulb. During the subsequent 3 weeks proteinuria increased further and renal function deteriorated until the patient was referred to our centre. Twenty-one days after the onset, laboratory tests at arrival were as follows: hemoglobin: 9.6 gr/dL; platelet count: 115.000/mm^3^; plasma albumin: 3.2 gr/dL; serum creatinine: 2.7 gr/dL; LDH: 484 IU/L; haptoglobin: undetectable; cholesterol 234 mg/dL; urinary protein/urinary creatinine: 5.1 mg/mg.

Given the clinical picture of TMA, functional and genetic tests for complement dysregulation were requested, and eculizumab treatment was immediately started (900 mg followed by a second dose after seven days).

**Figure 1:**
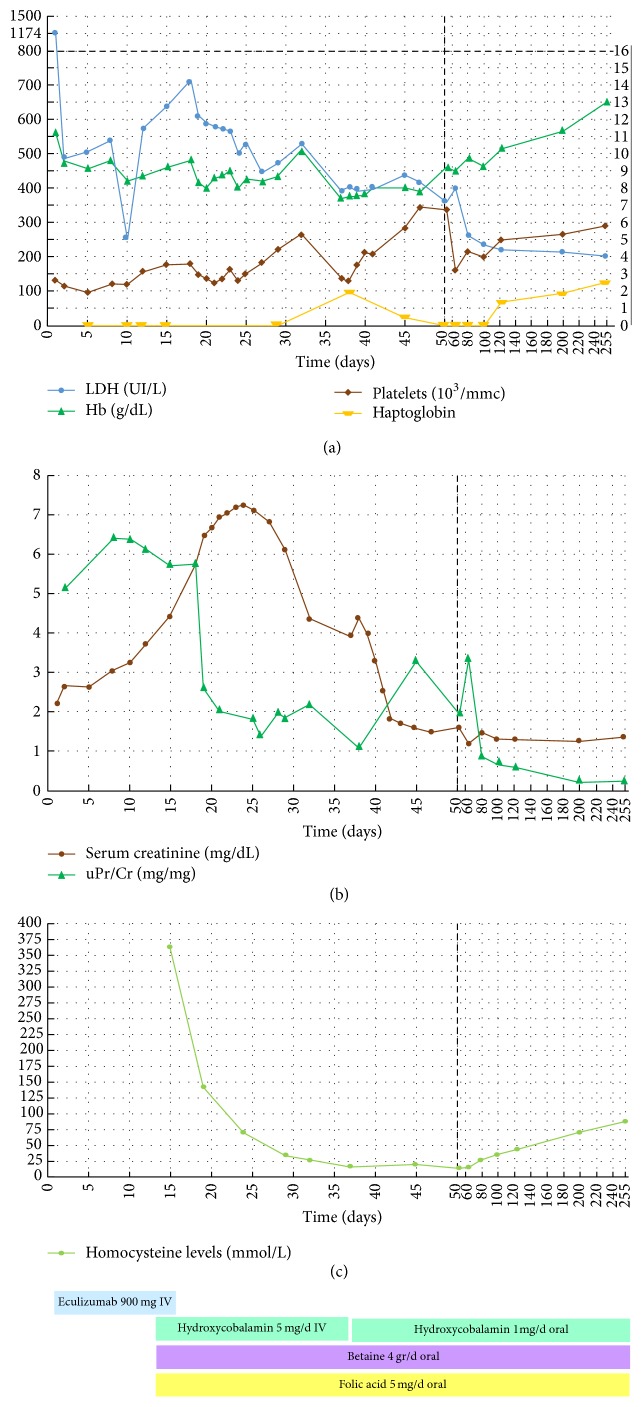
Laboratory relevant to TMA activity. (a) Hematological parameters: LDH (UI/L); Hb (g/dL); platelets (10^3^/mmc); (b) renal parameters: serum creatinine (mg/dL); uPr/Cr (mg/mg); (c) homocysteine levels (mmol/L).

Despite efficient complement inhibition (AP_50_: 1%) and a normalised platelet count (180,000/mm^3^), the hemolysis persisted (haptoglobin < 20 mg/dL) and kidney function continued to decline reaching a peak serum creatinine level of 7.2 mg/dL. For these reasons and for the normal level of complement C3 (93 mg/dL), laboratory tests were extended to rule out less frequent inherited causes of TMA. Hyperhomocysteinemia (364 mMol/L, normal range < 15.4) and hypomethioninemia (8 uMol/L, normal range 15–20 uMol/L) and increased urinary excretion of methylmalonic acid (20 mMol/mol, normal range < 2 mMol/mol) were detected. Since these metabolic alterations strongly suggested a cblC disease, the specific treatment with intravenous hydroxycobalamin (5 mg/day), betaine (4 g/day), and folic acid (5 mg/day) was started on the fourteenth day. The first evidence of a response was observed as early as 4 days after starting the specific treatment with a clear-cut reduction in the urinary protein/urinary creatinine ratio from 6.4 to 2.6 mg/mg (Figure 1). In the meantime, complement dysregulation was ruled out by molecular analysis of the relevant genes* (CFH, CFH-related, CFI, CFB, MCP, C3)* and by the normal titre of anti-CFH antibodies. HIV, antinuclear, and anticardiolipin antibodies and ADAMTS13 were also normal. Molecular analysis of the* MMACHC* gene definitively confirmed the diagnosis of cblC by identifying the known causative c.271dupA (p.Arg91Lysfs^*∗*^14) and c.388T>C (p.Tyr130His) mutations. The p.Tyr130His has been previously described in compound heterozygous state with c.481C>T (p.Arg161^*∗*^) mutation in a patient with onset of disease at 17 years. Since p.Tyr130 maps in the conserved cobalamin binding motif (122-HXXGX_126–154_GG_156_), the p.Tyr130His mutation may affect clb binding or protein structural integrity and it could be associated with a late onset phenotype.

Renal function gradually normalised during the following six weeks, as also the other TMA laboratory parameters, including proteinuria (Figure 1). Thirty-five days after admission, and while improving, the patient presented two episodes of generalised seizure, which were attributed to posterior reversible encephalopathy syndrome on the basis of magnetic resonance imaging.

The patient is currently well, and his hypertension only requires two drugs (atenolol and ramipril) and he is undergoing a maintenance treatment with oral hydroxycobalamin (1 mg once daily), betaine (4 g once daily), and folic acid (5 mg once daily).

## 3. Discussion

Both Shiga toxin-related and atypical HUS are unrare causes of acute kidney injury in children, but cblC disorder is responsible for fewer than 1% of TMA cases and is characterised by presentation very early in life (usually at an age of <1 year).

Our case underlines the importance of including homocysteine in the initial diagnostic work-up of all nephrotic patients beyond the typical age of idiopathic nephrotic syndrome of infancy (<6 years) as well as all cases of aHUS to screen for intracellular cobalamin metabolism disorders as these disorders are not limited to infancy.

Although most cases of aHUS are accompanied by various degrees of proteinuria due to endothelial damage at glomerular level, the development of overt nephrotic syndrome at the time of presentation is not common and may be misleading. However, it can be useful to mention that the loss of urinary proteins is not the only mechanism responsible for reduced albumin concentration in this setting: the disease is systemic (all of the microvasculature can be damaged); therefore widespread endothelial protein leakage takes place.

Although the pathogenetic mechanism of the endothelial damage in cblC-related TMA is very different from that of aHUS associated with complement dysregulation, a certain degree of complement involvement has been hypothesised also in other forms of TMA. In this regard, the partial but unequivocable response to eculizumab seems to provide indirect evidence that complement is involved in the mechanism of damage.

In conclusion, we strongly recommend including plasma homocysteine determination in the diagnostic work-up of any patient with nephrotic syndrome and/or with TMA in order to ensure timely diagnosis and effective treatment.
